# Risky-Driving-Image Recognition Based on Visual Attention Mechanism and Deep Learning

**DOI:** 10.3390/s22155868

**Published:** 2022-08-05

**Authors:** Wei Song, Guangde Zhang

**Affiliations:** School of Automobile and Traffic Engineering, Wuhan University of Science and Technology, Wuhan 430081, China

**Keywords:** risky driving, image recognition, deep learning, visual attention

## Abstract

Risky driving behavior seriously affects the driver’s ability to react, execute and judge, which is one of the major causes of traffic accidents. The timely and accurate identification of the driving status of drivers is particularly important, since drivers can quickly adjust their driving status to avoid safety accidents. In order to further improve the identification accuracy, this paper proposes a risky-driving image-recognition system based on the visual attention mechanism and deep-learning technology to identify four types of driving status images including normal driving, driving while smoking, driving while drinking and driving while talking. With reference to ResNet, we build four deep-learning models with different depths and embed the proposed visual attention blocks into the image-classification model. The experimental results indicate that the classification accuracy of the ResNet models with lower depth can exceed the ResNet models with higher depth by embedding the visual attention modules, while there is no significant change in model complexity, which could improve the model recognition accuracy without reducing the recognition efficiency.

## 1. Introduction

Motor vehicles have become an important means of transportation for the daily travel and cargo transportation of residents. Their present possession and annual increase show an explosive growth trend, which unavoidably causes an increasing number of traffic safety problems and accidents. Therefore, facing the development background of the above era, how to reduce the probability of traffic safety problems and improve the traffic safety factor has become a common concern of scholars. Risky driving is one of the essential factors leading to traffic safety problems. It causes the driver to have less control over the vehicle, which in turn leads to the driver being unable to perform normal car maneuvers, such as steering, gear shifting, and deceleration [1,2,3].

Statistical results indicate that more than 75% of traffic accidents and traffic safety problems are closely related to irregular driving and risky driving behaviors [4]. For example, during the driving process, calling, drinking and smoking can affect driver attention, making them unable to focus on the driving conditions ahead and the environment around the motor vehicle, which may directly lead to the occurrence of safety accidents. Therefore, it is important to improve the detection capacity of the driver’s driving status, and the timely identification and correction of risky driving behaviors can avoid traffic safety problems to the greatest extent [5]. At this stage, a large number of scholars have carried out experimental research on the detection of risky driving and have achieved relatively excellent performance. Among them, the early risky-driving-detection systems were mainly based on vehicle driving information, driver physiological signals or driver facial characteristics, and they also achieved relatively stable detection accuracy supported by accurate sensor devices. However, the traditional risky-driving-detection system still has some application problems, such as a slow detection efficiency, complex detection scheme and difficult application deployment.

With the further development of electronic imaging technology and the continuous innovation of computer intelligence technology, image-classification technology based on machine vision has flourished and has been applied to production tasks in various fields. Among them, deep-learning technology, as one of the hottest intelligent research directions in recent years, has shown extremely outstanding achievements in the field of image recognition and classification by automatically extracting features from input images using convolutional neural networks (CNNs). Therefore, deep-learning-based image-recognition and classification techniques have been applied to many fields such as medicine [6], machinery [7], and agriculture [8], and the different models have also been tested, experimented, and applied by researchers in the fields of automotive image recognition and driver status detection.

Based on deep-learning techniques, Alotaibi et al. proposed a distracted-behavior-detection system based on residual modules and recurrent neural networks (RNNs), and the experiment results proved that the method has high classification accuracy in the driver distracted-driving image-classification task [9]. Fusing in-vehicle sensor data with vision data, Furkan et al. proposed a system based on a CNN and migration-learning techniques applied it to hazardous driving condition detection and achieved a 96% detection accuracy on the test dataset [10]. To detect driver driving behaviors, Xing et al. designed a driver-activity-recognition system based on deep convolutional neural networks (CNNs) to detect seven common driving behaviors, compared the classification performance of three networks, AlexNet, GoogLeNet and ResNet50, and AlexNet was relatively better in the detection test [11]. In addition, different scholars have experimented, tested the performance of various types of deep-learning networks in risky-driving-image-classification tasks, and applied the related techniques to practical detection scenarios [12,13,14,15,16].

However, they focused on the risky driving behavior recognition accuracy of the existing deep-learning model, and did not consider the collaborative optimization of recognition accuracy and efficiency to reduce the difficulty of the deployment of the recognition system. As you know, the complexity of a model determines the speed of its response, and we can reduce that complexity by reducing the depth of the model. Therefore, this paper explores the classification accuracy of deep-learning models at different depths, and introduces the visual attention module to further enhance each classification model, so as to explore risky-driving-image-recognition models with low model complexity and high classification accuracy, which can provide guidance for model selection in different application scenarios. The key contributions of this work are:

(1) Taking the driver’s risky-driving images as the research object, including four categories of images: normal driving, driving and drinking, driving and smoking, and driving and calling, this paper proposes four different visual attention modules and builds ResNet image-classification models of different depths. 

(2) This experiment embeds the proposed four visual attention modules into the ResNet models to explore the classification performance. 

(3) This experiment introduces the Grad-CAM algorithm for visual analysis to observe the influence of the visual attention modules for feature extraction in risky-driving images.

The rest of this paper is organized as follows: Section 2 discusses the structure of base convolutional neural networks, pooling strategies, visual attention modules, and the data augmentation technique. Section 3 indicates the experimental results and discussions, and Section 4 concludes the paper with a summary and future research directions.

## 2. Methodology

This section describes various techniques involved in the visual attention mechanism and deep-learning-based risky-driving-image-classification systems, mainly containing convolutional neural networks, ResNet architecture, different pooling operation schemes, different types of visual attention modules, and data-augmentation techniques.

### 2.1. Convolutional Neural Networks & ResNet

**Figure 1:**
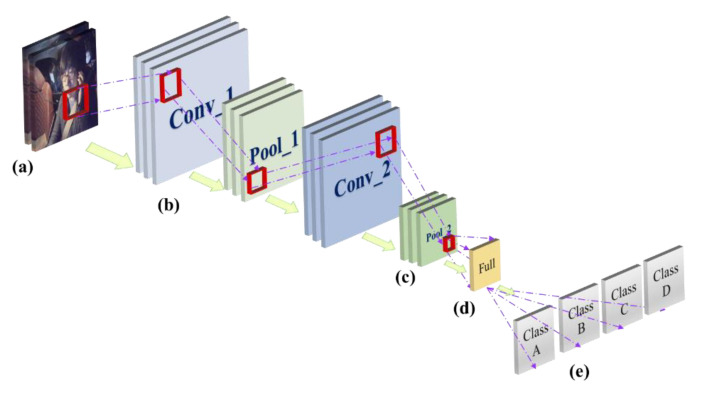
Convolutional neural network architecture: (**a**) input layer; (**b**) convolutional layer; (**c**) pooling layer; (**d**) fully connected layer; (**e**) classification layer.

Convolution neural networks (CNNs) are a kind of feed-forward neural network with a deep structure and convolution calculation, which has strong learning capability and uses a convolution layer structure to classify input information shift invariant [17]. The basic CNNs consist of five structures: thr input layer, convolutional layer, pooling layer, fully connected layer and classification layer. The CNN network architecture is shown in Figure 1.

**Figure 2:**
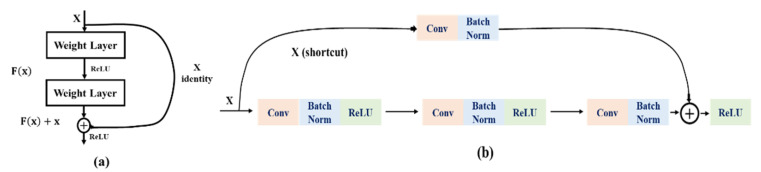
Residual module: (**a**) residual module operation process; (**b**) shortcut connection.

ResNet [18] is a class of networks designed to solve the gradient explosion and the overfitting problems during the model-training phase as the network deepens. The purpose of the residual module (Figure 2a) is to add the features extracted from the front to back layers of the model, and by using the shortcut connection (Figure 2b), ResNet effectively solves the problems of network gradient explosion and overfitting during the training process. At the same time, by introducing the batch-normalization (BN) layers, ResNet speeds up the network training speed and convergence stability. Due to the application advantages of the ResNet, this experiment selects a different-depth ResNet as the guiding architecture to complete the risky-driving-image-classification tasks.

### 2.2. Pooling Operation & Different Pooling Strategies

**Figure 3:**
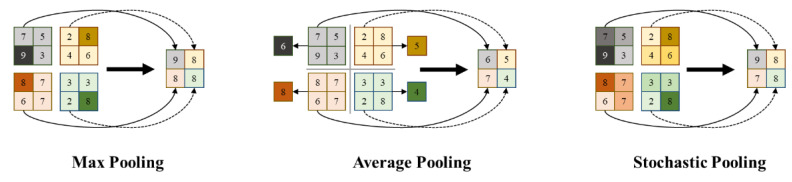
Different pooling strategies in convolutional neural networks.

The pooling operation is one of the most important processing units in the CNN models, which plays the role of extracting representative features for captured image features, and therefore is also called the sub-sampling or down-sampling operation. After the pooling operation, the dimension of the output feature is effectively reduced, which is helpful for reducing the network training parameters and preventing overfitting. In the CNN architecture, the common pooling strategies include max pooling, average pooling and stochastic pooling, as shown in Figure 3.

For different types of image-classification tasks, different pooling strategies can focus on preserving different image features, such as texture, contour, background or other types of features in the input feature maps, and researchers can select different pooling strategies to optimize the CNN models for a specific task. However, it is worth knowing that the single pooling strategy often results in the loss of useful feature extraction. For instance, max pooling discards all non-maximum values in the pooling kernel, while average pooling fails to retain the maximum feature values, and stochastic pooling does not focus on the retention of features in a specific direction. Therefore, the single pooling strategy also limits the classification performance of the CNN models, and needs to be compensated for and solved by the optimization methods.

### 2.3. Visual Attention Module Design

To solve the problem of feature loss caused by using a single pooling strategy and to improve the classification performance of deep-learning models in risky-driving-image-classification tasks, this paper proposes to incorporate visual attention mechanisms into risky-driving-image-classification models, and this section mainly illustrates the four visual attention module design schemes.

#### 2.3.1. Squeeze and Excitation Visual Attention Block (SE Block)

**Figure 4:**
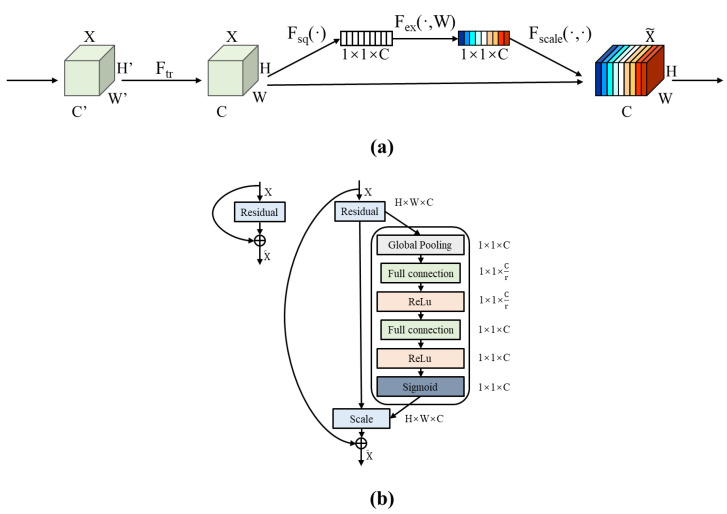
Structure and embedding scheme of the SE block: (**a**) SE block structure; (**b**) SE block application scheme in ResNet.

The squeeze and excitation visual attention block (SE block) was firstly proposed by Hu et al. in SE Net [19], which adds the visual attention mechanism to the CNN model in the channel direction to obtain more channel feature information, and the structure of the SE block is shown in Figure 4a.

The SE block mainly contains three processing processes: squeeze, excitation and scale. The output of the previous layer is the processing object, and a 1 × 1 convolution operation is performed first to obtain the feature map.

(1) $$u_{c}=v_{c}*X=\sum_{S=1}^{c^{\prime }}v_{c}^{s}*x^{s}$$

where $v_{c}$ represents the number of parameters of the c-th filter, the X is the input image, ∗ represents the convolution operation process, and $u_{c}$ is the output feature map.

Afterwards, the SE block will use the convolutional output to perform squeeze, excitation and scale operations in sequence, where the squeeze process is implemented as a global average pooling operation, that is, each feature channel of the feature map is compressed and characterizes the global distribution of responses over the feature channels; the excitation process is implemented by using a fully connected layer. The result after excitation is subjected to another fully connected operation to achieve feature dimensionality recovery, and the sigmoid activation function is used to obtain a weight value between 0 and 1. This process allows the CNN model to effectively learn the nonlinear interactions and nonreciprocal relationships between channels, and ensures the attention enhancement of multiple channels. Finally, the output values of the excitation processing are subjected to a reweight process that is used to weight the normalized weights to the features of each channel, also known as scale, which is weighted to the previous features channel by channel through the dot product. Through the SE block operation, the CNN model is effectively enhanced for feature extraction in the channel direction, and the SE block can be flexibly embedded in the residual branch of the ResNet model, as shown in Figure 4b.

#### 2.3.2. Channel Visual Attention Block & Spatial Visual Attention Block (CA Block & SA Block)

**Figure 5:**
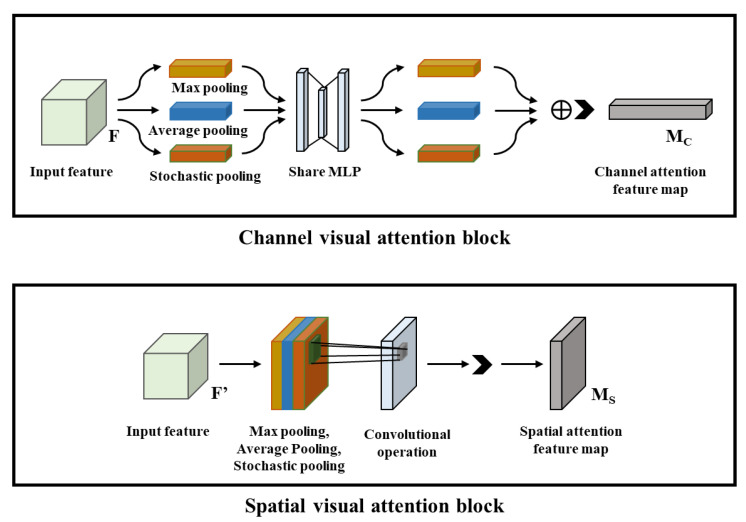
Structure of the proposed channel attention block and spatial attention block.

Referring to the design idea of the SE visual attention block, Woo et al. proposed two new visual attention blocks, the channel attention module (CA block) and the spatial attention module (SA block), for spatial direction and channel direction, respectively [20], which further improve the feature-extraction ability and classification performance of the CNN image-classification model. The structure details of the CA block and SA block are shown in Figure 5.

In the CNN image-classification models, the CA block and SA block focus on performing visual attention tasks in different ways, where the CA block focuses on computing the intrinsic relationships between individual channels, while the SA block focuses on the intrinsic relationships of feature maps at the spatial level.

On the one hand, in the CA block, it performs the max pooling, average pooling and stochastic pooling operations on the input feature map F to simultaneously obtain the texture, contour and background information of the input image and enhance the model robustness. Finally, the computation result will be sent to an MLP shared network, which will sum the corresponding elements of the three different feature maps and output the channel attention feature map, so the CNN model not only obtains the reduced dimensionality of the output feature images in the convolutional layer, but also retains more comprehensive image features. On the other hand, in the SA block, the max pooling, average pooling and stochastic pooling are performed on the input feature maps in turn, and the results are obtained for feature concatenation. Then, the fused feature maps are subjected to a standard convolution operation to recover the feature dimension and output the spatial visual attention feature map, so the SA block can efficiently help the CNN model solve the problem of “which regions are important and which regions are minor” in the input image. In addition, both the CA block and the SA block can be flexibly deployed in the ResNet, and their embedding schemes are similar to those of the SE block.

#### 2.3.3. Mixed Visual Attention Block (MA Block)

**Figure 6:**
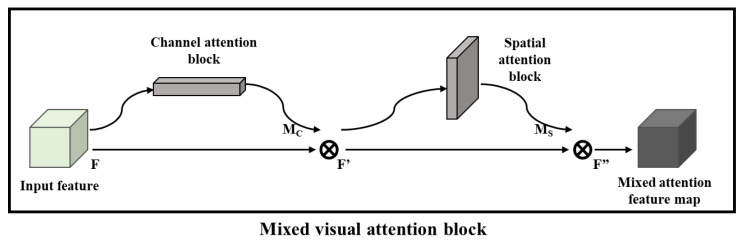
Structure of the proposed mixed visual attention block.

In the process of exploring the use of visual attention mechanisms in CNN models, Woo et al. found that there is still space for the upward improvement of CNN image-classification models, so they proposed a mixed visual attention block that combines the use of two types of visual attention blocks to improve the feature-extraction and image-classification performance of deep-learning models, as shown in Figure 6. Meanwhile, through experiments, Woo et al. pointed out that setting the CA block in front and the SA block in the back has a more significant performance on the model enhancement, and the increase in computational complexity contributed by this MA block to the CNN model is relatively small. In addition, the embedding method of the MA block in the ResNet model is consistent with the deployment of the SE block, CA block and SA block, which indicates the high application flexibility of the MA block.

### 2.4. Data-Augmentation Technology

With the deepening of the deep-learning model and the increase in the model complexity, training a new, deep and large CNN image-classification model needs to be supported by a large amount of labeled image data, and an insufficient amount of image data will directly lead to overfitting and accuracy bottlenecks during the training phase. Besides, as a relatively new research area, there are relatively few public datasets and insufficient image data for the risky-driving-image-classification task. In addition, the acquisition of risky-driving images requires a professional camera at a fixed position on the driver’s side of the motor vehicle, which has relatively strict requirements for imaging equipment and shooting environments, which also increases the difficulty of acquiring risky-driving images and preparing data sets.

One solution to the above problem is the data-augmentation (DA) technology, which is now widely used by researchers to obtain training data that can be used for deep-learning models. Specifically, the classic DA methods includes rotating, flipping, scaling, increasing contrast, adding Gaussian noise, and many other forms. Among them, rotation processing rotates the original training image by a certain angle; flipping inverts the original image horizontally or vertically; scaling enlarges or shrinks the original image by a certain proportion; increasing contrast changes the saturation (S) and value (V) of the original image in the HSV color space; adding Gaussian noise randomly perturbs each pixel RGB in the original image. Therefore, by using the above classic DA methods, researchers can quickly and efficiently expand the training image dataset for their CNNs models, which in turn alleviates the problems of overfitting and unbalanced data volume between groups during the training phase.

## 3. Results and Discussion

### 3.1. Experiment Data Processing & Dataset Preparation

For the task of monitoring the driver’s driving status, this experiment selected the normal driving status and three risky-driving-status images as the experiment object, among which, the three risky-driving-status images include smoking, drinking and calling. During driving status, drivers’ behaviors of smoking, drinking and calling will seriously distract drivers’ attention and reduce their response speed to emergencies, so when the above scenarios occur, the probability of drivers causing potential safety hazards or traffic accidents will also increase significantly. Therefore, the above risky-driving situations should be avoided as much as possible in the actual driving process.

**Figure 7:**
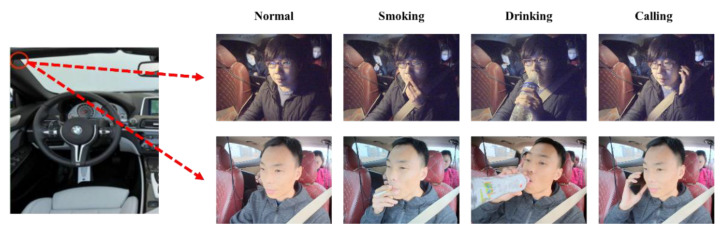
Camera deployment location and four categories of driving status.

The experimental data were collected by a professional image data acquisition company, the camera was deployed in the left side of the motor vehicle above the A-pillar, which can clearly capture the driver’s driving status images, and selected two images in each category as an example. The camera deployment position and the acquired images are shown in Figure 7.

At the beginning stage of the dataset preparation, this experiment adopted an 8:1:1 ratio to divide the training set, test set and validation set, respectively. In addition, in order to avoid the problems of insufficient training data and an uneven data volume between different categories of images, this experiment used the DA technology to expand the training set. The data volume of each category after the DA process is shown in Table 1, in which the training sets of normal, smoking, drinking and calling are 2403, 2420, 2416 and 2407, respectively, totaling 9646, the test set is 298, 293, 291 and 299, totaling 1181, and the validation set is 280, totaling 1120.

### 3.2. Model-Building Details and Experiment Setting

In order to explore the performance of different-depth CNNs and the proposed visual attention blocks for risky-driving image classification, four deep-learning image-classification models with different depths were built with reference to the ResNet, which are ResNet18 with 18 layers, ResNet34 with 34 layers, ResNet50 with 50 layers, and ResNet101 with 101 layers. After that, the four visual attention blocks (SE block, CA block, SA block, and MA block) were embedded in the different-depth The model-building details and the embedding details of the visual attention blocks are shown in Table 2. By comparing the above CNN models, this experiment will systematically explore the performance of deep-learning-based image-classification technology in the field of safety driving detection.

In the model-building, platform-deployment and testing phases of this experiment, the details of its experimental environment and application platform are shown in Table 3. Among them, this experiment selected the SGD model optimizer, a learning rate of 1 × 10^−4^, a momentum of 0.95, a discard rate of 0.5, a loss function of category cross-entropy loss, and in the deployment of the attention module, its decay rate was 16 and the pooling kernel size was 7 × 7. Meanwhile, in order to improve the model-training efficiency, this experiment used ReduceLROnPlateau and EarlyStopping algorithms, where the monitor of ReduceLROnPlateau was validation loss, the decay learning rate (factor) was 0.5, and the patience was 4. In EarlyStopping, its monitor was validation loss, the Min_delta was 0, and the patience was 10. In addition, the models were built based on the Keras toolbox in the Python 3.7 environment, the training epoch was 400 with a batch size of 32, and all models were trained in Nvidia RTX 2080Ti and CUDA10.1, cudnn7.3.1 environment.

### 3.3. Experiment Result Comparison and Analysis

#### 3.3.1. Model Comparison and Evaluation

To fully evaluate and compare the performance of the deep-learning models embedded with attention blocks in risky-driving-image-classification tasks, and to explore the application performance between different visual attention blocks, this experiment collected the training accuracy, training loss, validation accuracy, and validation loss of 20 different ResNet models, as shown in Table 4. Among them, training accuracy and training loss were used to evaluate the model-training status, and validation accuracy and validation loss were used to evaluate the model classification performance. Meanwhile, the number of calculation parameters of each model was calculated to evaluate the training difficulty of the models and to observe the increase in training parameters and the training difficulty due to embedding visual attention blocks.

This experiment compares the base ResNet models with different depths without the visual attention mechanism. The results indicate that the classification accuracy is positively correlated with the depth of the model, and the model complexity, that is, the number of calculation parameters, is negatively correlated with the depth of the model. Among them, ResNet101 achieved 92.73% classification accuracy with 45.13 M parameters in the risky-driving-image dataset; while the recognition accuracy was improved, the model was also more complex.

After that, we compared the ResNet models of each depth embedded with visual attention blocks, and the results indicate that the visual attention modules can enhance the recognition accuracy of the ResNet model to varying degrees, but they do not significantly increase the number of model parameters. Among them, the ResNet models embedded with the MA block improved the validation accuracy to the greatest extent, and the improvement degrees of ResNet18_Mixed, ResNet34_Mixed, ResNet50_Mixed and ResNet101_Mixed were 4.93%, 4.39%, 3.63% and 4.45%, respectively. 

It is worth noting that the classification accuracy of the ResNet models with lower depths can exceed the ResNet models with higher depths by embedding the visual attention modules. For instance, the validation accuracy of ResNet50_Mixed was 96.52%, while that of ResNet101 was 93.73%, and the number of parameter in ResNet101 was 60% more than ResNet50_Mixed. Therefore, we can greatly improve the recognition accuracy of the ResNet model by embedding a visual attention module, but the recognition efficiency will not be affected, which is of great significance to the practical application and popularization of this technology.

#### 3.3.2. Confusion Matrices Analysis

**Figure 8:**
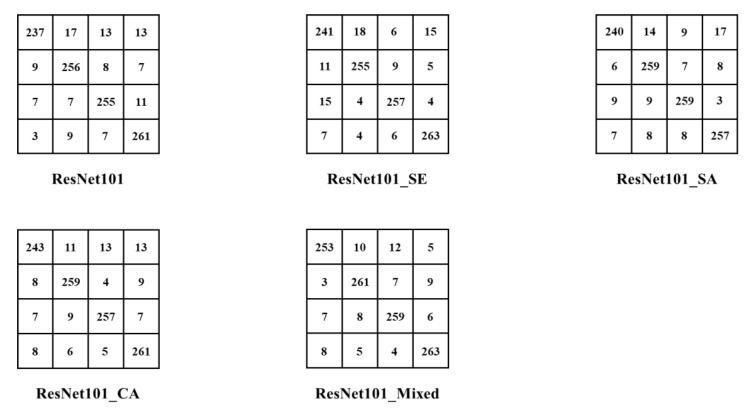
Confusion matrices of ResNet101 models with visual attention blocks to further demonstrate the classification performance.

To further demonstrate the classification performance of the proposed ResNet model embedded with attention blocks in the risky-driving-image dataset, the confusion-matrices-evaluation tool was introduced in this experiment [21,22]. Taking the ResNet101 and the ResNet101 variant model with different visual attention blocks as examples, the confusion matrices of the above models in the risky-driving-image test set are shown in Figure 8.

On the whole, the ResNet101 models with attention blocks had a lower misclassification rate compared with the base ResNet101 model, in which the number of misclassifications for ResNet101_SE, ResNet101_SA, ResNet101_CA, and ResNet101_Mixed we’re 104, 105, 100, and 84, respectively, while the number of misclassification images of the base ResNet101 model reached 111, which indicates that embedding the visual attention module to the ResNet model can effectively improve the classification performance of the CNN model for risky-driving images.

When analyzing the misjudgment rate of different categories, the results indicate that the misclassification of normal-driving images was relatively high in the four categories, while the misclassification of smoking, drinking, and calling we’re more similar in the three categories. In analyzing the reasons for the above results, we believe that the normal-driving images do not have obvious characteristics, and their driving actions have partial similarity to the remaining three risky-driving images, which to some extent causes the model misclassification.

#### 3.3.3. Grad-CAM Visualization Analysis

**Figure 9:**
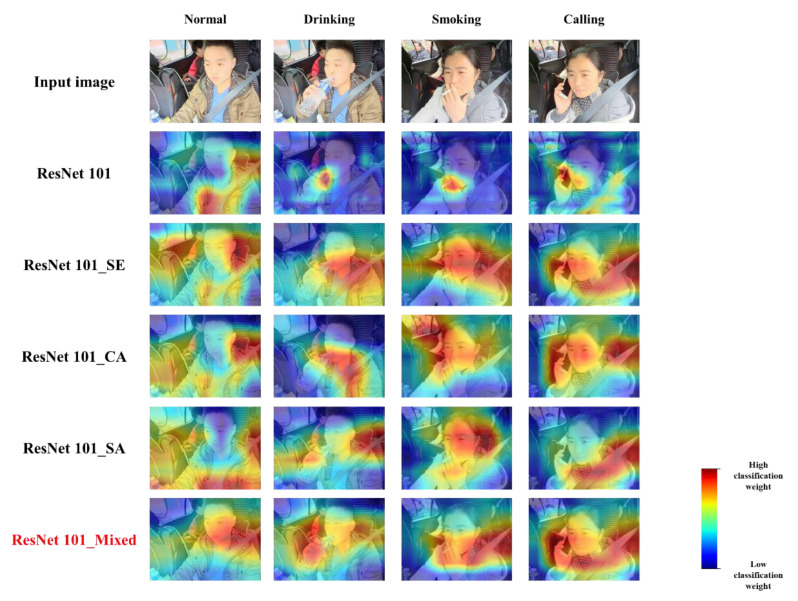
Grad-CAM maps of ResNet101 models with visual attention blocks to observe the changes in the magnitude and distribution of classification weights.

In order to observe the changes in the magnitude and distribution of the classification weights caused by embedding visual attention blocks more intuitively, taking the different-depth ResNet101 models and variant ResNet101 models as examples, one image of each category in the risky-driving-image dataset was selected for Grad-CAM visualization [23], and the visualization results are shown in Figure 9.

On the one hand, in the overall trend, the visualization results show that after embedding attention blocks to the ResNet101 model, the model extracts more image feature information in the input image, as the regions that account for the weights are relatively increased, which indicates the improved feature extraction ability of the ResNet model. On the other hand, when analyzing Grad-CAM map of the ResNet101 with different attention blocks, the experimental results show that the distribution and size of the weights occupied by some of the focal regions in the three risky-driving category images increase to some extent. For example, the weight of the water bottle and drinking-action region in the driving-while-drinking image, the weight of the cigarette stick and smoking-action region in the driving-while-smoking image, and the weight of the cell phone and talking-action region in the driving-while-talking image, which represents the main direction of the features extracted by the visual attention blocks.

## 4. Conclusions

In order to further improve the performance of the deep-learning image-classification model in the risky-driving-detection task, this paper proposes a solution of embedding visual attention blocks into the deep-learning framework to improve the feature-extraction ability and classification performance. Through the model comparison and evaluation, it is worth noting that the classification accuracy of ResNet models with lower depths can exceed the ResNet models with higher depths by embedding the visual attention modules, while there is no significant change in model complexity. Therefore, we can greatly improve the recognition accuracy of the ResNet model by embedding the visual attention module, but the recognition efficiency will not be affected, which is of great significance to the practical application and popularization of this technology. Moreover, the results of the confusion matrices analysis and Grad-CAM visualization analysis confirm the superiority of the proposed model.

In future studies, we will further expand the amount of dangerous-driving-scene recognition and image data, optimize the configuration of the visual attention module, and carry out practical applications and optimization on the basis of improving the accuracy and efficiency of recognition.

## Figures and Tables

**Table 1:** Data volume of risky-driving-image dataset.

	Normal	Smoking	Drinking	Calling	Total
Training set	2403	2420	2416	2407	9646
Valid set	298	293	291	299	1181
Test set	280	280	280	280	1120

**Table 2:** Model structure setting and the insertion position of attention blocks.

Layers	Output Size	Res Net18	Res Net34	Res Net 50	Res Net101
Conv 01	112 × 112	7×7, 64, stride 2			
		Attention block			
Conv 02	56 × 56	3×3 max pool, stride 2			
		$\left[\begin{array}{c} 3\times 3,\;64\\ 3\times 3,\;64 \end{array}\right]\times 2$	$\left[\begin{array}{c} 3\times 3,\;64\\ 3\times 3,\;64 \end{array}\right]\times 3$	$\left[\begin{array}{c} 1\times 1,\;64\\ 3\times 3,\;64\\ 1\times 1,\;256 \end{array}\right]\times 3$	$\left[\begin{array}{c} 1\times 1,\;64\\ 3\times 3,\;64\\ 1\times 1,\;256 \end{array}\right]\times 3$
		Attention block			
Conv 03	28 × 28	$\left[\begin{array}{c} 3\times 3,\;128\\ 3\times 3,\;128 \end{array}\right]\times 2$	$\left[\begin{array}{c} 3\times 3,\;128\\ 3\times 3,\;128 \end{array}\right]\times 4$	$\left[\begin{array}{c} 1\times 1,\;128\\ 3\times 3,\;128\\ 1\times 1,\;512 \end{array}\right]\times 4$	$\left[\begin{array}{c} 1\times 1,\;128\\ 3\times 3,\;128\\ 1\times 1,\;512 \end{array}\right]\times 4$
		Attention block			
Conv 04	14 × 14	$\left[\begin{array}{c} 3\times 3,\;256\\ 3\times 3,\;256 \end{array}\right]\times 2$	$\left[\begin{array}{c} 3\times 3,\;256\\ 3\times 3,\;256 \end{array}\right]\times 6$	$\left[\begin{array}{c} 1\times 1,\;256\\ 3\times 3,\;256\\ 1\times 1,\;1024 \end{array}\right]\times 6$	$\left[\begin{array}{c} 1\times 1,\;256\\ 3\times 3,\;256\\ 1\times 1,\;1024 \end{array}\right]\times 23$
		Attention block			
Conv 05	7 × 7	$\left[\begin{array}{c} 3\times 3,\;512\\ 3\times 3,\;512 \end{array}\right]\times 2$	$\left[\begin{array}{c} 3\times 3,\;512\\ 3\times 3,\;512 \end{array}\right]\times 3$	$\left[\begin{array}{c} 1\times 1,\;512\\ 3\times 3,\;512\\ 1\times 1,\;2048 \end{array}\right]\times 3$	$\left[\begin{array}{c} 1\times 1,\;512\\ 3\times 3,\;512\\ 1\times 1,\;2048 \end{array}\right]\times 3$
		Attention block			
Fully connected	1 × 1	Average pool, softmax			

**Table 3:** Model-training hyperparameter setting.

	Type	Setting
Basic setup	Optimizer	SGD
	Learning rate	1 × 10^−4^
	Momentum	0.95
	Dropout	0.5
	Loss function	categorical_crossentropy
Attention block setting	Decay rate	16
	Pooling kernel	7 × 7
Training setting	Batch size	16
	Epoch	400
ReduceLROnPlateau	Monitor	Validation loss
	Factor	0.5
	Patience	5
EarlyStopping	Monitor	Validation loss
	Min_delta	0
	Patience	10
Training environment	GPU	Nvidia RTX 2080Ti
	Platform	Python 3.7
	Toolbox	Keras

**Table 4:** Recognition accuracy and efficiency evaluation of ResNet models with different depths by embedding visual attention blocks (especially the mixed visual attention block, greatly improving the recognition accuracy while not affecting the recognition efficiency).

	Train Accuracy	Train Loss	Valid Accuracy	Valid Loss	Parameters
Res Net 18	91.42%	1.3640	88.25%	1.6353	11.75 M
Res Net 18_SE	95.62%	0.5830	92.26%	1.1715	11.82 M
Res Net 18_CA	94.48%	0.6733	92.12%	1.1863	11.82 M
Res Net 18_SA	94.83%	0.6098	92.04%	1.1924	11.82 M
Res Net 18_Mixed	95.75%	0.5873	93.18%	1.1056	11.82 M
Res Net 34	93.34%	1.0892	90.78%	1.3528	21.85 M
Res Net 34_SE	95.74%	0.5914	93.21%	1.0968	22.31 M
Res Net 34_CA	93.85%	1.0008	93.94%	1.0819	22.31 M
Res Net 34_SA	95.41%	0.5948	93.16%	1.1056	22.31 M
Res Net 34_Mixed	97.90%	0.4551	95.17%	0.6242	22.32 M
Res Net 50	94.36%	0.6278	92.89%	1.1363	25.68 M
Res Net 50_SE	96.02%	0.5642	94.95%	0.5954	28.21 M
Res Net 50_CA	95.95%	0.5772	94.43%	0.6163	28.21 M
Res Net 50_SA	95.45%	0.5824	94.15%	0.6451	28.21 M
Res Net 50_Mixed	98.29%	0.3484	96.52%	0.5263	28.22 M
Res Net 101	95.89%	0.5364	93.73%	1.1396	45.13 M
Res Net 101_SE	97.81%	0.4428	95.94%	0.5779	49.91 M
Res Net 101_CA	96.71.%	0.5284	95.25%	0.5994	49.91 M
Res Net 101_SA	96.96%	0.5189	95.14%	0.6148	49.91 M
Res Net 101_Mixed	99.28%	0.0932	98.18%	0.3595	49.91 M

## Data Availability

Not applicable.
